# Climate Change and Health in British Columbia: Projected Impacts and a Proposed Agenda for Adaptation Research and Policy

**DOI:** 10.3390/ijerph7031018

**Published:** 2010-03-11

**Authors:** Aleck Ostry, Malcolm Ogborn, Kate L Bassil, Tim K Takaro, Diana M Allen

**Affiliations:** 1 Department of Geography, University of Victoria, Victoria, BC, V8W 3R4, Canada; 2 University of Northern British Columbia, Prince George, BC, V2N 4Z9, Canada; E-Mail: ogborn@unbc.ca; 3 Department of Health Sciences, Simon Fraser University, Burnaby, BC, V5A 1S6, Canada; E-Mails: kate_bassil@sfu.ca (K.L.B.); ttakaro@sfu.ca (T.T.); 4 Department of Earth Sciences, Simon Fraser University, Burnaby, BC, V5A 1S6, Canada; E-Mail: dallen@sfu.ca

**Keywords:** climate change, British Columbia, health, adaptation

## Abstract

This is a case study describing how climate change may affect the health of British Columbians and to suggest a way forward to promote health and policy research, and adaptation to these changes. After reviewing the limited evidence of the impacts of climate change on human health we have developed five principles to guide the development of research and policy to better predict future impacts of climate change on health and to enhance adaptation to these change in BC. We suggest that, with some modification, these principles will be useful to policy makers in other jurisdictions.

## 1. Introduction

Disentangling the complex indirect pathways between climate change and health is a challenging research endeavour requiring the development of new inter-disciplinary academic partnerships and cross-industry, governmental, and professional alliances. It is also essential to create a research infrastructure to provide more accurate baseline and continuing data on the future impacts of climate change on human health. Given the complex way in which climate change will act in concert with other socio-economic and environmental factors it is best to approach the study of climate change and health using a social determinants of health framework.

The purpose of this paper is to outline how climate change is likely to affect the health of British Columbians and to suggest a way forward to promote health, policy research, and adaptation to these changes. Although this study was conducted in the Western Canadian province of British Columbia (BC) we feel that the insights from this case will be useful for other jurisdictions grappling with questions of research and policy in relation to climate change and health.

In the first section of the paper, the basic conceptual frameworks used to investigate climate changes and health are introduced. In the second section, we describe the health status of British Columbians with a particular focus on the health status of rural residents of the province as it is quite likely that rural and remote communities will be among the first to experience the impacts of climate change as it unfolds in the province. In the third section we outline the evidence linking climate change to adverse health outcomes. Because there is limited evidence available for BC (a situation also fairly typical for other regions in many developed nations), we extrapolate from this limited evidence to suggest possible impacts of climate change on the health of British Columbians. In the fourth section, we outline a “made in BC” set of principles and priorities for a research and policy agenda to improve adaptation to the health impacts of climate change. In the final section of the paper we suggest ways in which this BC case may apply to other jurisdictions.

## 2. Models Used to Investigate the Impacts of Climate Change on Health

Several conceptual models have been developed in order to frame research on the impacts of climate change on health [1–12]. In many of these models, climate change is conceptualized as impacting human health directly or indirectly [3,4]. Most climate change research has been conducted on the direct effects of climate on health. Since 1970, the WHO conservatively estimates 150,000 deaths per annum the direct effects, mainly from heat waves and flooding, due to climate change [13,14]. These events have significant immediate public health impacts and, from a research and policy standpoint, offer the potential to demonstrate a direct link with climate change.

Indirect effects on health arise from the sustained impact of altered climate which cause changes to ecosystems and which, in turn, impact human health through various pathways [15]. For example, rises in temperature may alter living conditions for animal and plant vectors of diseases with subsequent, very difficult to predict, impacts on human health [8]. Other models posit more complex pathways from climate and ecosystem transformation to adverse health outcomes including, for example, impacts of social dislocation and migration and increased food insecurity that might arise from these broad changes to ecosystems [9,16].

Other researchers have taken a more ecological and place-based approach to conceptualizing the potential impacts of climate change on health [5,11,12,17–23]. For example, in a comprehensive review of findings from the United Nations Intergovernmental Panel on Climate Change, the National Research Council, the World Health Organization, and the United Nations Environment Program, Patz & Kovats (2002), posit that places at particular risk from the adverse effects of climate change on human health are those facing concurrent environmental and socioeconomic stress [11]. They, and others, point to the need to systematically identify these “hotspots” in order to identify communities, as well as sub-populations within these communities, particularly at risk and to begin strategies of adaptation [24]. Hess *et al.* (2008) also take a place-based approach to better understanding the potential for climate change to affect health noting that public health threats related to climate change are location specific [5]. They propose the systematic identification of places where human populations and critical infrastructure are at risk and where exposure to climactic extremes are likely in order to develop risk management strategies to best cope with emerging impacts of climate change on human health. As well, Hess *et al.* (2008) show how place attachment is important in climate change adaptation, as it fosters both increased awareness of the ecological shifts seen with climate change as well as motivation to adapt in order to maintain effective place attachment relationships. This may be important in BC, particularly among the province’s aboriginal peoples, who tend to have strong place attachment that may be helpful in climate change adaptation targeting these vulnerable groups.

Finally, according to Woodward *et al.* (1998), the magnitude of the effects of climate change on health will be a function both of the nature and magnitude of climatic changes and the *vulnerability* of the affected populations [12]. *Thus, it is essential that research on human health effects of climate change must, not only grapple with the intensity and pace of climate change but also with the location and extent of vulnerability of human populations most likely exposed to climate change* [3,4].

## 3. The Health Status of British Columbians

British Columbia is Canada’s westernmost province. It is a ruggedly mountainous place, with extensive forest cover and limited arable land. The population of approximately 4.5 million people is concentrated around the city of Vancouver located in the extreme south-west of the province and, to a lesser extent, in the south-central Okanagan Valley and on Southern Vancouver Island.

An investigation into the health status of rural compared to urban Canadians using mortality data from 1996 to 2001 and the 2001 Canadian Community Health Survey (CCHS) demonstrated that rural Canadians were systematically worse off than urban Canadians. Residents of rural regions across Canada were also much poorer than people living in urban places. As well, people in rural Canada had lower levels of education, higher rates of unemployment, and higher dependency ratios (*i.e.*, high populations of seniors and/or children and youths relative to the working age population). Rural regions attract fewer immigrants and also have a higher proportion of Aboriginal residents than most urban regions. The study also showed that while death rates for most cancers were lower for rural Canadians they experienced much lower life expectancy, higher all-cause mortality, higher prevalence of arthritis, greater mortality from injury and accidents, suicides, diabetes, cardio-vascular disease and they had a higher prevalence of smoking and obesity relative to urban Canadians. The greatest differences between urban and rural Canadians was in the much higher prevalence of arthritis/rheumatism in rural regions and the much greater mortality from injury and accidents (particularly from motor vehicle accidents), and from suicide [25].

While BC is one of the most healthy jurisdictions in the world, as in the rest of the country, there are large disparities in the socio-economic and health status of rural and urban residents of the province. For example, approximately 25% to 40% of British Columbians live in predominantly rural (depending on the definition of rurality) or northern areas [26]. These regions of the province have been (particularly since the early 1980s), experiencing and responding to extensive and rapid economic change including the new socio-environmental threat posed by the Mountain Pine Beetle (MPB) infestation of the forests surrounding many communities in the central regions of the province. Globalization and concomitant social changes (e.g., industrial downsizing and restructuring in the forestry sector [27,28] and the restructuring associated with welfare and healthcare reform), have resulted in depopulation of rural and remote towns as well as in the dramatic alteration of community social fabric and in the working and living conditions of individuals in BC’s rural and northern communities placing many of them under duress as they attempt, often with fewer resources than are available in urban communities, to respond to multiple emerging and ongoing social and environmental challenges [29].

Besides this heightened socio-economic and environmental vulnerability, residents of northern regions of the province are less healthy on average than residents of urban communities. For example, according to the 2005 Canadian Community Health Survey, approximately 20 percent of residents of BC’s northern regions were obese and approximately 25 percent smoked, compared to, respectively, 8 and 12 percent in the city of Vancouver, the major urban center located in the south-west corner of the province [30]. This health behaviour gap is also found between urban and rural communities located in the southern part of the province. For example, in 2005, smoking rates averaged 20 to 25 percent in the south east corner of the province (a region with no major urban centers) and obesity rates in this region of the province were approximately twice those found in Vancouver [30].

These urban/rural and urban/northern differences in self-reported health behaviours are also evident for objectively measured health outcomes. As an example of the seriousness and extent of this northern/urban health gap in BC, the average age standardized mortality rate between 1997 and 2000 was 6.4 deaths per 1,000 in the Fraser Health Authority (largely urban and located in the south west corner of the province) compared to 8.4 per 1,000 in the Northern Health Authority (largely rural and covering the northern half of the province) [31,32]. The almost one third difference in the mortality experience of residents in these two health authorities is akin to what one might find comparing the current mortality experience between Canada and a much less developed much poorer nation such as Jamaica.

This health divide is also found when considering life expectancy. Residents in Vancouver currently have life expectancies that are among the highest in the world (approximately 82 years) but residents of the Northern Health Authority, on average, have life expectancies which are 4 or 5 years less than this. Thinking about the gap in self-reported health status, morbidity, mortality, and life expectancy temporally, it is as if current residents of some rural and northern communities have health status that was typical of urban British Columbians 40 years ago [31].

While health vulnerability is high for people living in rural and remote regions of the province there are also vulnerable sub-populations in the province’s towns and cities. While it is not completely clear how these vulnerable urban populations will be affected in BC by future climate changes we know from studies, mainly in the United States and Northern Europe, that the urban poor are particularly vulnerable to adverse impacts arising from acute heat exposure during prolonged heat waves [33].

For example, heat exposure has been studied in the United States and Northern Europe mainly in relation to urban heat waves. Cities are disproportionately affected by heat because of the urban heat island effect. In major urban areas increases in temperature of up to 11°C warmer than in surrounding areas have been observed [34]. The impact of the urban heat island effect is magnified in the very young and very old [35] and for the urban elderly living in poverty [36–39]. And, during the 1995 heat wave in Chicago, there were 465 heat-related deaths and those over age 65 accounted for 70 percent of these [40].

Some individuals living in poor urban areas and some living in remote and rural regions of the province are vulnerable both socio-economically and in terms of their health status. As well, in the case of rural and remote communities, there is some evidence that their social fabric may be less able to adapt to economic and environmental disruption relative to urban communities [29]. This heightened vulnerability in rural and remote communities is, as will be shown in the next section, an important issue to consider in relation to potential impacts of climate change on the health of British Columbians.

## 4. What will be the Impact of Climate Change on the Health of British Columbians?

British Columbia is a province with a classic wet temperate climate along the coast and Vancouver Island. Most of central BC has a typical continental climate with a cold winter and a warm summer with precipitation distributed relatively evenly through the year. The exception is the Okanagan Valley in the south centre of the province. This region is very dry all year round. The north of the province is characterized by a sub-arctic climate. The province is traversed by large mountain ranges and major river systems, including the Columbia, Fraser, Liard, and Stikine rivers. The geography, hydrology, and climate of the province is complex and diverse making the development of predictive models linking future climate change and human health particularly challenging.

### 4.1. The Mountain Pine Beetle Infestation in British Columbia

A factor of importance in considering the potential impacts of climate change on human health in BC is the major infestation of the province’s extensive pine forests by the Mountain Pine Beetle (MPB) [41]. The Mountain Pine Beetle is endemic to North America’s pine forests. Warmer winters in conjunction with more aggressive harvesting of pine trees in the province appear to have triggered widespread infestation beginning in the early 1990s. While the MPB epidemic in BC’s pine and mixed forests has now peaked, it has caused such extreme devastation that the damage in the central and central northern regions of the province is visible from space. (This MPB infestation is also occurring south of BC in the United States and is beginning, to spread into the Rocky Mountain region, an area normally with little MPB presence because of its colder climate). MPB infestations normally die out in the winter with the onset of cold weather. Recent warming of winters in the province has meant that this natural control of MPB infestation is interrupted. Thus, the extensive MPB epidemic in the province may be, at least partly, the result of a secular change in the climate [42,43].

Impacts of climate change on health due to fire and flood could be considerable given the complicating factor of widespread forest kill due to MPB infestation. MPB killed trees retain their dead needles for as much as 3 or 4 years after initial infestation. During this period, these trees are at higher risk for fire than healthy trees. As well, dead standing trees, in combination with increased precipitation due to climate change, especially during spring melt, may intensify spring water run-off patterns increasing the potential for flooding and drinking water contamination [44–46]. It is therefore likely that increased fire and flood will occur, particularly in the extensive zones of the province affected by MPB [47].

People living in communities located in the interior and northern interior of the province are therefore vulnerable, not only in terms of their reduced economic and health resilience, but also because of the direct and indirect impacts of this major biological disaster, likely itself, directly due to the effects of a changing climate.

## 4.2. Physical Changes that will Influence Health Include Greater Exposure to Fire and Flood and Increased Exposure to Heat and Air Pollution

Exposure to fire, flood, heat and increased air pollution are all likely pathways to increased health risk for residents in both urban and rural regions of BC as climate change accelerates. Cities are disproportionately affected by heat because of the urban heat island affect [34] which is magnified in the very young and very old [4,33,35,38–40,48] and for the urban elderly living in poverty [36,37,49]. Research in New Orleans after Hurricane Katrina demonstrated that low income, less well-educated non-white residents of the city were most likely to sustain adverse health outcomes [50] a result also noted from the impacts of severe storms and flooding in other nations [51]. Both chronic and acute health impacts of floods in BC communities will likely be greater for vulnerable sub-populations.

While parts of the province will get wetter, some regions are at risk of shortages of both surface and ground water [44,46,52]. Retreating glaciers, declining snowpack, earlier spring melt, higher evapotranspiration, shifts in the timing and amount of precipitation, and prolonged drought are expected to limit water supply during peak demand periods, and lead to increased competition among water users [53–57]. Declining stream flow and groundwater levels, which also affect water quality, coupled with increased runoff due to extreme events, may lead to greater impacts from water-borne pathogens and other contaminants as well as increased sediment in drinking water [45,58,59]. And, as noted in the previous section, impacts of climate change on health due to fire and flood could be considerable given the complicating factor of widespread forest kill due to MPB infestation.

Forest fires are projected to become more frequent and severe in Western Canada [60,61] and in the Western United States [62]. We know also from recent fires in the town of Barriere and Kelowna (although we don’t know for sure that these two particular fires were the direct result of climate change) that there are direct and often severe impacts on health [62]. For example, an investigation during a three week period during the 2003 fire season near Kelowna showed an increases in physician visits for respiratory diseases of between 46 and 78% above aggregate rates for the same weeks in the previous ten years [63]. As well, according to Nelson Ames, Medical officer of health for the Kootenays Region in discussing the impact of the Kelowna fire, “cases of mental and physical exhaustion have already surfaced, and some people are exhibiting symptoms of post-traumatic stress disorder” [64].

Air pollution has a well known adverse impact on respiratory health [65,66]. Air pollution in conjunction with temperature increase may potentiate adverse health impacts particularly in urban regions and especially by enhancing exposure to ozone [67–69]. Air pollution/temperature interactions could be further potentiated by increased particulate exposure due to wood smoke exposure from more severe and frequent forest fires [70].

It is possible that climate change in temperate places, like BC, will produce relatively benign conditions for agricultural production in the short and medium term as temperatures warm in formerly northern cold regions [16,71]. There is some speculation (but no real evidence) that northern BC (especially the vast Peace River region) may experience increased agricultural yields for some crops. If this were the case, it is possible that as climate change evolves in BC, its impacts on human health *via* its impacts on food security may be moderate or even positive at least in the short and medium term.

### 4.3. Biological Changes will Arise when Temperatures and Precipitation Increase

Water-borne diseases may increase as a result of increased precipitation and flooding [72]. Many respiratory pathogens such as influenza exhibit winter seasonality. There is limited evidence of the potential for increased prevalence of influenza and other respiratory illnesses with the arrival of El Nino (wetter) weather patterns in the United States [73,74]. Food-borne gastroenteritis, particularly illnesses related to Campylobacter and Salmonella, currently exhibit a summertime pattern of occurrence [75]. Rises in temperature will likely see growth in the prevalence of these types of illness in human populations in BC [76].

Rise in temperature may alter living conditions for animal and plant vectors of diseases [8,77–79]. The higher average rainfall and temperatures and earlier onset of spring predicted for BC could result in prolonged transmission cycles for some vectors of human disease [80,81]. And, higher temperatures and precipitation may extend the northern ranges of currently established vector borne disease such as Lyme disease which is well established in the south-west corner of the province [82].

New fungal pathogens from warmer and wetter climates may find the local soil ecology and climate more welcoming with climate change. For example, Cryptococcus gatti, a tropical fungus, first appeared in 1999 on the South East coast of Vancouver Island an emergence that may be due to changing climatic conditions [83]. This fungus has caused over 100 cases of human illness and appears to be spreading from Vancouver Island to south-west corner of the mainland [84].

### 4.4. Socio-Economic Changes

Most of the discrepancy in chronic and physical and mental illness seen across cultural, economic, ethnic, and geographic dimensions arises as a result of social rather than biological determinants of health [47]. BC’s vulnerable communities, and the vulnerable sub-populations living in them, will likely be more heavily impacted by climate change than more robust communities and sub-populations [12,85]. There is a need, therefore, to focus climate change research on vulnerable populations and communities. It will be particularly important to determine the impact of climate change among vulnerable rural and remote communities in the province and within these communities it will be important also to focus on the health impacts on children and the elderly as these sub-populations will be the most vulnerable to the adverse health impacts of climate change [86].

The importance of a focus of climate change research and policy on vulnerable rural and remote communities in BC cannot be overstated for several reasons. First, approximately 25 percent of the province’s population lives in rural and remote places [87]. Second, the socio-economic conditions in most rural and remote places in BC are systematically worse (and in some cases much worse) than they are for urban British Columbians [88]. Third, the health status of rural British Columbians is systematically worse, across a broad range of health outcomes than for urban citizens [89]. Fourth, of all rural and remote communities in BC, Aboriginal communities have by far the worse socio-economic and health profiles so that research on these communities, especially those most affected by climate change is essential. Fifth, rural and remote places in central and northern BC are already facing a severe environmental disaster, namely infestation of pine trees by the Mountain Pine Beetle. This infestation may, when it is finally over, result in the death of over 90 percent of pine trees in the province [90]. Finally, these remote and often isolated communities are often more exposed than other communities to extreme weather events and located further from services to relieve their impact if and when they occur.

## 5. A Climate Change and Health Research and Policy Agenda for BC

Frumkin *et al.* (2008) have proposed a public health approach to climate change based on the essential public health services emphasizing the necessity of developing and coordinating classical public health, non-governmental and government services to deal with the complex health and infrastructural problems that will arise as climate change begins to impact human health [91]. As well, McMichael, in propounding a basic epidemiological approach to investigations of the impacts of climate change on health has suggested the importance of retrospective studies of associations between climate variability and change for various health outcomes using established datasets as well as prospective surveillance of likely health impacts occurring currently as a result of climate change for which there are few if any other explanations. Finally, he has suggested scenario-based integrated assessment modeling to project climate change associated health impacts [92]. Based in part on these approaches, as well as on the unique circumstances facing British Columbia, we suggest five principles to guide development of research and policy to enhance adaptation to the health impacts of climate change in BC.

### 5.1. Basic Research Is to Develop “Made In BC” Models and Infrastructure for Use in Climate Change and Health Investigations

It is necessary to obtain better local meteorological data as climate monitoring networks are not adequate to measure BC’s highly variable climate. Next it is necessary to develop models that apply accurate regional climate change to regional topography, hydrology and ecological systems, to better predict flood and fire risk, surface and ground water quality change, and the introduction and spread of new and existing vector-borne and other infectious illnesses. Presently, accurate climate change projections are not possible because the models are not well developed and also because future emissions trajectories are also not yet clear. As well, it is imperative to conduct field testing to confirm the predictive power, particularly of hydrological models, developed in order to determine their utility. In the meantime, there are reasonable regional estimates of past current and climate change projections available that account for topography for the province [93,94].

Given the high prevalence of chronic illness, particularly cancer, depression, respiratory, and heart disease in BC, relatively small disease exacerbations associate with climate change could result in substantial increases in absolute disease burdens. Research is required to elucidate these indirect pathways with these outcomes. It is essential to have good quality longitudinal health data sets available for research as climate change and health studies require at least 30 years worth of data [95]. BC is uniquely positioned to be a leader in climate change and health research because of the BC Linked Health Database (BCLHDB). This database consists of person-specific longitudinal records on all residents of British Columbia. The BCLHDB contains files on all births, physician services utilization, hospital discharges, drug prescriptions, long term care services, enrolment in the mental health client registry, Worker Compensation claims, cancer incidence, and fact and cause of death, from 1985 to the present. The records are housed at the University of British Columbia’s Center for Health Services and Policy Research (CHSPR) and are managed jointly by the Center, the University, and the Ministry of Health. The BCLHDB is managed according to the provisions of British Columbia’s Freedom on Information and Protection of Privacy Act. Each file is stored separately but has been indexed with an individual service-recipient-specific code so that the records of groups of individuals can be linked across files for specific research projects [96].

The data base has been extensively utilized over the past 15 years for complex longitudinal investigations of various health outcomes, in several different populations, and in relation to a variety of environmental and occupational exposures [97–100]. These data can be further developed and ways found to facilitate their availability for climate change and health research.

### 5.2. Need to Focus Climate Change and Health Research on Vulnerable Communities

Action is required to enhance the adaptive capacity of the most vulnerable regions and the most vulnerable people within regions. An emerging research agenda must focus on understanding regional and community resilience, particularly among vulnerable sub-populations in order to build on these. Research on social capital of communities, flexibility and innovation of government, private sector and non-profit agencies, and programs is needed. In short the role of collective coordination and action in facilitating adaptation is important [13].

In the BC context, the potential for adverse impacts due to climate change will be particularly high in rural and remote Aboriginal communities where the combination of highly socio-economically vulnerable and health vulnerable populations and long distances from health and emergency services will potentiate any adverse impacts arising due to climate change. Other vulnerable communities in BC include some non-Aboriginal rural and remote communities as well as many poor urban ones.

### 5.3. Development of Adaptation Policy Requires Education and Mobilization of the Public As Well As Health *and* Other Professionals

Promotion of local adaptation policies requires public and professional buy-in and greater awareness by the public. In terms of the latter, it will be essential to investigate the perceived risk of climate change in the population [101–104]. The effectiveness of strategies for adapting to climate change depend on the social acceptability of options for adaptation, the institutional constraints on adaptation, and the place of adaptation in the wiser landscape of economic development [13]. Promotion of local adaptation policies requires public and professional buy-in. This requires fostering strong collaborations with health authorities, municipalities and others responsible for individual and public health, and developing strong policies promoting knowledge transfer from researchers to stakeholders. Most importantly, adaptation strategies must be customized for local conditions and populations.

Mobilization of the public and government and non-government stakeholders may not be easy even in relation to climate change. For example, Leiserowitz (2006) [102] has shown in a population-based survey of Americans, that most individuals feel that climate change is a moderate danger likely to impact people and places that are far away and of little personal importance. Lorenzoni and Pidgeon (2006) [103] have also shown that these attitudes are largely shared by Britons and suggests that engaging the public on this issue is challenging. These attitudes are likely quite common among Canadians.

Engagement is key to moving individuals and community leaders forward in developing adaptive strategies to deal with climate change as it unfolds because it is only when individuals in communities feel vulnerable to the impacts of climate change and understand that their community livelihood and their health may be threatened that they will be moved to make individual changes and press their communities for adaptive strategies. A key area of research to develop is in risk perception of climate change impacts of health in order to understand regional variation in these perceptions as these will be a marker of the potential for successful adaptation polices to be enacted at the local level.

### 5.4. Adaptation to Climate Change Will Require Development of New Collaborations across Government, Regional Health Authorities, and Non-Government Agencies As Well As an Enhanced Surveillance Role for *Public* Health Authorities

The approach outlined by Frumkin *et al.*, is key here [91]. Public health surveillance, emergency preparedness, and research functions must be bolstered in order to take on a stronger leadership role in climate change and health adaptation policy development and implementation. As well, the public health system in BC has a key leadership role to take in these collaborations because it has the credibility, the medical and managerial skills (ranging from disease surveillance to disaster response planning), as well as experience and engagement across ministries and sectors and at the provincial and local levels, to coordinate policies to adapt to the health impacts of climate change.

Also, several public health principles point to well established public health approaches to climate change. First, what public health professionals understand as primary prevention would be mitigation. Secondary and tertiary prevention would be adaptation (*i.e.*, efforts to anticipate and prepare for effects of climate change thereby reducing the associated health burden). The idea that steps to protect public health from threats of climate change cannot await full scientific certainty is also consistent with prevailing public health practice and management for other illnesses [91].

### 5.5. Climate Change Will Strain the Provincial Health Service in Uneven Fashion Across Regions. To Ensure *Equitable* Access to Health Services Better Understanding of the Future Impacts of Climate Change on Health Services Is Required

BC already has major challenges in insuring equitable access to health services across its communities. As climate change is likely to produce changes in demography, changes in the health problems they experience, and widen existing disparities, the pressure to innovate in service delivery, particularly in vulnerable communities will increase.

The BC Linked Health Database will be a key research resource for monitoring change in access and equitable availability to health services across regions. While this data base must be further developed to improve data on impacts of climate change on health services in the immediate future it provides a strong research foundation for beginning baseline studies of the health status of residents of rural and remote communities on the front lines of climate change.

## 6. Conclusions

We would argue that the five principles that emerge from this case study are useful for other jurisdictions beginning the process of determining research needs and policy action to determine and adapt to potential impacts of climate change on health. Although some of the particulars of this case study are clearly unique to the province. For example, BC has the BC Linked Health Database, an unusually powerful longitudinal database of health outcomes that will not be available to researchers in other places. As well, the Mountain Pine Beetle infestation occurring in the province is an event more or less unique to BC.

The following six principles derived from this case study of BC may be useful for developing a climate change/health research agenda in other developed nations. First, basic research that “fits” local circumstances, including as a first step, the gathering of reliable regional climactic information in a way that is linkable to health outcomes (for illnesses with high prevalence locally) is essential. Second, climate change will aggravate existing health disparities so that research and adaptive policy must be directed towards those people and communities currently with the greatest deficits in health. Third, this means that vulnerability assessment (*i.e.*, identification of “hotspots” as in methods outlined by Hess *et al.*, among others [5]) must be a priority in order to proactively both identify vulnerable communities and sub-populations. Fourth, adaptation requires mobilization of the public as well as health and other professionals especially in and near hotspots identified through rigorous vulnerability assessments. Fifth, adaptation to climate change will require development of new partnerships across government, and non-government agencies as well as an enhanced surveillance role for public health authorities. Finally, climate change will strain health service in uneven fashion across regions so that planning to ensure equitable access to health services is required.
